# Bilateral primary hyperplastic persistent vitreous: report of two cases

**DOI:** 10.3205/oc000169

**Published:** 2020-10-09

**Authors:** Neha Singh, Siddharth Agrawal, Pallavi Mishra

**Affiliations:** 1 Department of Ophthalmology, King George Medical University, Lucknow, Uttar Pradesh, India

**Keywords:** PHPV, leucocoria, Peters’ anomaly, B-scan

## Abstract

Primary hyperplastic persistent vitreous (PHPV) or persistent fetal vasculature is a rare clinical entity that presents with leucocoria, microphthalmos, and cataract. It is mostly unilateral. Here we present a report of two cases of bilateral PHPV. One of the patients had associated Peters’ anomaly. The entity closely mimics retinoblastoma and should be kept as a differential diagnosis of bilateral leucocoria. Examination under anesthesia, ultrasound B-scan, and aqueous lactate dehydrogenase levels helped us reach the diagnosis and differentiate it from the more serious entity retinoblastoma.

## Introduction

Primary hyperplastic persistent vitreous (PHPV) is the result of the anomalous development of the primary vitreous as it persists into the time of formation of the secondary vitreous [1]. It is a rare clinical entity that presents with leucocoria, microphthalmos, and cataract [2]. It can cause complications like tractional retinal detachment, secondary glaucoma, strabismus, and amblyopia [2]. It is mostly unilateral, and bilateral PHPV is rare [3]. Here we present a report of two cases of bilateral PHPV. One of the patients had associated Peters’ anomaly. The entity closely mimics retinoblastoma and should be kept as a differential diagnosis of bilateral leucocoria.

## Case description

### Case 1

A male child of 1 month presented with the inability to open the left eye from birth. The child had a full-term normal vaginal delivery with a birth weight of 3,500 grams. On clinical examination, the left eye appeared smaller and there was a bilateral white reflex raising the clinical suspicion of retinoblastoma.

A detailed examination was done under general anaesthesia. In the right eye, horizontal and vertical corneal diameters were 9 mm and 7 mm, respectively; and in the left eye 7 mm and 6 mm, respectively. IOP was 8 mm Hg and the anterior chamber was shallow in both eyes. Fundus examination showed membrane extending from the optic disc in the right eye. In the left eye, no posterior details could be visualized due to total cataract. Aqueous samples were collected and sent for Lactate dehydrogenase (LDH) levels which came to be normal.

**Figure 1:**
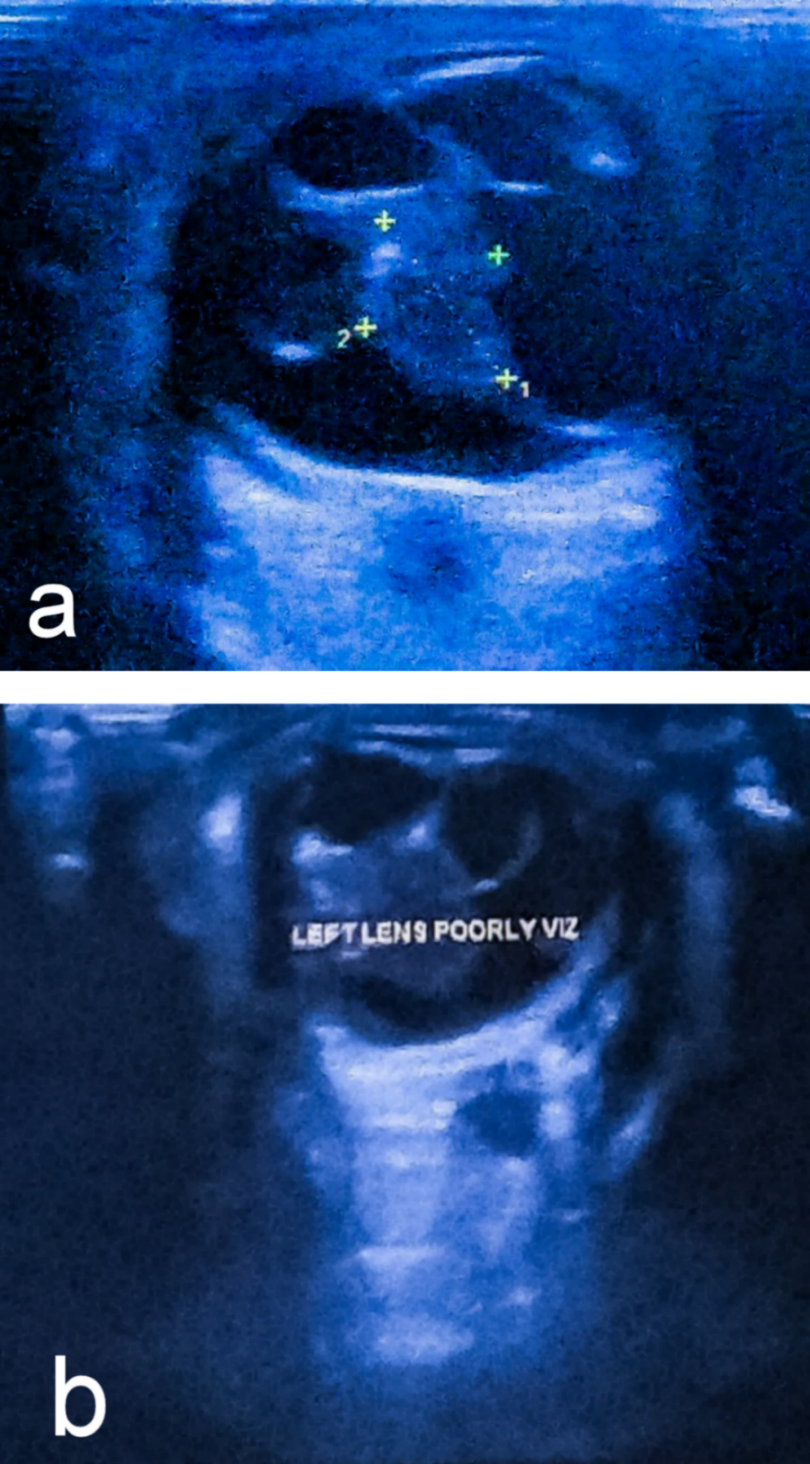
Ultrasound B-scan of (a) the right eye and (b) the left eye (b) showing an echogenic membrane extending from the retina to the posterior surface of the lens

**Figure 2:**
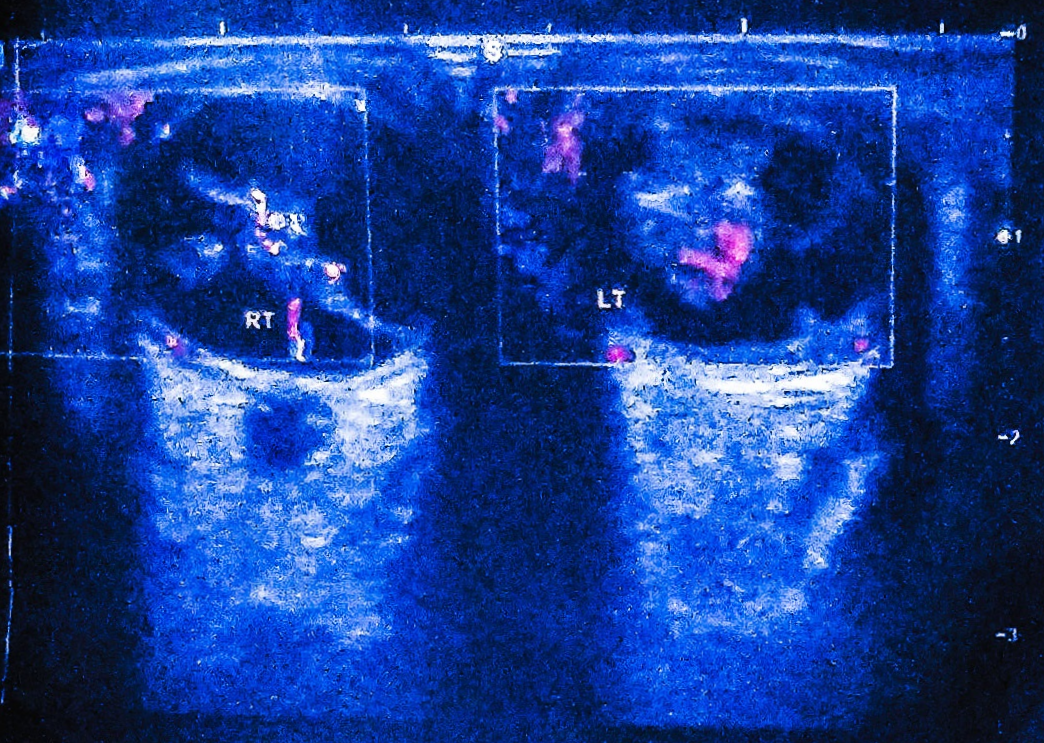
Color Doppler showing vascularity

A B-scan revealed an ill-defined hyperechoic soft tissue lesion of size 10.3×7.4 mm in the right and 12.6×6.6 mm in the left posterior chamber (Figure 1 (Fig. 1)). The lesion also shows hyper reflection echogenic linear strands with few debris. The bilateral lens appeared normal but the left lens capsule was thickened and compressed by the lesion anteriorly. On color Doppler the lesion showed mild vascularity (Figure 2 (Fig. 2)). Based on these clinical findings and investigations, the diagnosis of bilateral PHPV was made. The patient’s parents were informed about the pathology, the visual prognosis and possibility of subsequent complications. The parents refused any further intervention.

### Case 2

**Figure 3:**
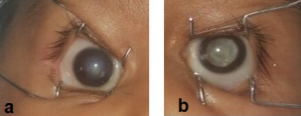
(a) Central corneal opacity in the right eye, (b) cataract in the left eye

A three-month-old otherwise healthy male was referred to our center with suspicion of bilateral retinoblastoma. Birth history was not significant. On ophthalmological examination, central corneal opacity was noted in the right eye. White pupillary reflex was present in both eyes. A detailed examination under general anesthesia revealed horizontal and vertical corneal diameters of 9 mm and 8 mm respectively in both eyes. Central corneal opacity was noted in the right eye (Figure 3a (Fig. 3)). A clinical diagnosis of Peters’ anomaly was made in the right eye. Intraocular pressure was 10 mm Hg in both eyes. Fundus details could not be visualized due to media haze in both eyes. Aqueous samples were collected for LDH which came out to be normal.

**Figure 4:**
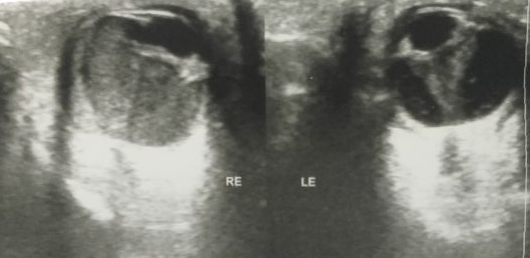
Ultrasound B-scan of the right eye (RE) and the left eye (LE)

A B-scan was done for posterior segment assessment (Figure 4 (Fig. 4)). It showed dense immobile echoes filling the posterior segment of the right eye and thickening of chorioretinal layers. No obvious calcifications were seen. The left eye showed echogenic membrane extending from the posterior surface of the lens to the optic nerve head. Based on these clinical findings and investigations, a diagnosis of bilateral PHPV with Peters’ anomaly in the right eye was made. The patient’s parents were made aware of the nature of the disease and associated complications. However, they were subsequently lost to follow-up.

## Discussion

PHPV is the result of the anomalous development of the primary vitreous as it persists into the time of formation of the secondary vitreous [1]. The primary vitreous is formed during the 1^st^ month of intrauterine life and starts regressing during the formation of the secondary vitreous at the 9^th^ week. By the end of the 3^rd^ month, the secondary vitreous fills most of the vitreous cavity, and the primary vitreous condenses into a narrow band (Cloquet’s canal) running from the optic nerve to the posterior aspect of the lens [1].

PHPV is sometimes further divided into subtypes. Anterior PHPV occurs when the remnant vascular stalk is seen attached to the back of the lens, but no longer extends back to the optic nerve [4]. Posterior PHPV occurs when the remnant vascular stalk is seen arising off the optic nerve but not reaching the lens and therefore not usually causing cataract. Posterior PHPV may be associated with developmental abnormalities of the optic nerve or surrounding retina. The surrounding retina can be scarred or even detached. If there is significant involvement of the optic nerve and/or retina, good vision may not be possible. Most often, patients have some element of both anterior and posterior PHPV [2].

Bilateral leucocoria in a child may be due to a number of causes, of which congenital cataract, retinoblastoma, retinopathy of prematurity (ROP), and retinal dysplasias are the most frequent [5]. Cataract is a common cause of leucocoria in children. It carries a good visual prognosis if not associated with any retinal pathology and when treated early enough. However, a primary congenital cataract was not suspected in our case as the cataracts in our patients were of the posterior capsular type. Moreover, a B-scan revealed pathology in the posterior segment.

PHPV is usually unilateral [5], [6]. Pollard [3], in his study of 83 cases of PHPV, reported only two cases (2.4%) that had bilateral PHPV. Bilateral presentations are commonly associated with systemic and syndromic associations such as trisomy 13, 15, or 18, Norrie’s disease and Warburg’s syndrome [7]. However, no systemic anomalies were present in our patients.

The differential diagnosis in our cases included retinoblastoma, ROP and PHPV. Normal gestational age, birth weight and uneventful birth history eliminated the possibility of retinopathy of prematurity. Presence of microphthalmia, cataract and normal intraocular pressure made the possibility of retinoblastoma less likely. It was supported by normal aqueous LDH levels, ultrasound B-scan and color Doppler.

One of our patients had Peters’ anomaly in the right eye. This association has been reported twice in the literature in the past. Matsubara et al. [8] described two cases with bilateral Peters’ anomaly and PHPV. They postulated that migratory disorder of neural-crest cells from 4 to 7 weeks of gestation was responsible for those ocular anomalies. Muslubas et al. [9] reported a case of unilateral PHPV with Peters’ anomaly and morning glory syndrome. They suggested the possibility of mutation in the PAX 6 gene for this association.

## Conclusion

To conclude, younger age at presentation, presence of microphthalmos, cataract and associated congenital ocular anomalies make the possibility of PHPV more likely in cases of bilateral leucocoria. Typical findings in ultrasonography, color Doppler and normal aqueous LDH levels help in differentiating PHPV from the more serious entity retinoblastoma.

## Notes

### Competing interests

The authors declare that they have no competing interests.
